# A conceptual change in crystallisation mechanisms of oxide materials from solutions in closed systems

**DOI:** 10.1038/s41598-020-75241-z

**Published:** 2020-10-27

**Authors:** Sibu C. Padmanabhan, Timothy W. Collins, Suresh C. Pillai, Declan E. McCormack, John M. Kelly, Justin D. Holmes, Michael A. Morris

**Affiliations:** 1 Advanced Materials and BioEngineering Research (AMBER) Centre, Trinity College Dublin, College Green, Dublin 2, Ireland; 2 School of Chemistry, Trinity College Dublin, College Green, Dublin 2, Ireland; 3 School of Chemistry, University College Cork, College Road, Cork, T12 YN60 Ireland; 4 Centre for Precision Engineering, Materials and Manufacturing Research (PEM), Institute of Technology Sligo, Ash Lane, Sligo, F91 YW50 Ireland; 5 Nanotechnology and Bio-Engineering Research Group, Department of Environmental Science, Institute of Technology Sligo, Ash Lane, Sligo, F91 YW50 Ireland; 6 School of Chemical and Pharmaceutical Sciences, Technical University Dublin, City Campus, Kevin Street, Dublin 8, D02 HW71 Ireland

**Keywords:** Inorganic chemistry, Materials chemistry, Chemical physics, Reaction kinetics and dynamics

## Abstract

Atomic and molecular level interactions in solutions dictate the structural and functional attributes of crystals. These features clearly dictate the properties of materials and their applicability in technologies. However, the microscopic phenomena of particle formation—nucleation and growth—in real systems are still not fully understood. Specifically, crystallisation occurring in closed systems are largely unproven. Combining coherent experimental data, we here demonstrate a fundamental nucleation-growth mechanism that occurs in a model zinc oxide system when particles are formed under continuous, rapid heating under closed reaction conditions. Defying all previous reports, we show that the nucleation commences only when the heating is terminated. A prenucleation clusters pathway is observed for nucleation, followed by crystallite assembly-growth. We show that the nucleation-growth processes result from temporal and dynamic activity of constituent ions and gaseous molecules in solution and by the irreversible expulsion of the dissolved gaseous molecules. We suggest that this nucleation process is generic to most closed systems that go through precipitation, and, therefore, important for the crystallisation of a variety of metal oxides, composites and minerals. We anticipate that the work may be a platform for future experimental and theoretical investigation promoting deeper understanding of the nucleation-growth phenomena of a variety of practical systems.

## Introduction

Nucleation and crystal growth mechanisms attract much interest because of their importance in science, technology and in many natural and biological processes^1–8^. Thus, there has been significant amount of experimental and modelling studies to understand these mechanisms in model systems such as atomic liquids, Lennard–Jones liquids, water, colloids, solutions, binary systems and natural gas hydrates^9–12^. Studies on real systems have also been reported^13–17^. Recent work has shown deviations from classical nucleation-growth models towards non-classical series of events involving ‘stable’, metastable or transient intermediates^18–21^. Oriented attachment and colloidal assembly growth have also been reported for many systems^22–27^. One theoretically and experimentally predicted pathway is through intermediate prenucleation clusters (PNCs)^14,15,19,20,27–29^. PNCs are three-dimensional (3D)-like arrangements of constituent atoms/ions formed in solution prior to nucleation, whose structural arrangement is related to the final crystal structure^15,30,31^. However, full understanding of the nucleation-growth phenomena remains remote and issues such as PNC stability and its transition into nuclei is unclear for many real systems. Despite advances in theoretical and modelling studies to enhance knowledge of the crystallisation process, accounting for all the aspects of real systems is extremely challenging. In this work, we provide the very significant finding that in a model ZnO system, nucleation does not occur during heating but rather when heating is terminated. Our findings reveal that the heating allows the system to reach a supersaturated state, wherein, temporally fluctuating hexagonally close packed-like spatial arrangements of Zn^2+^ and OH^−^ ions—PNCs—are formed, surrounded by loosely packed ions and species. When the heating is terminated, the temperature and pressure drop, and the dissolved gaseous molecules expel irreversibly from the system. This initiates the nucleation within the PNCs. Growth then follows by aggregate addition of the formed crystallites, driven by their inherent growth habit and the interfacial compatibility between them.

ZnO was selected for this study because it is a well-studied system for crystal growth from solution^32–44^. The biological and technological significance of ZnO underline its selection^45–48^. Synthesis was carried out by reacting aqueous solutions of 5 mM zinc nitrate (**1**) and 50 mM urea (**2**). For the rapid, continuous heating, a microwave (MW) reactor (CEM Technology Discover) was used. A MW power of 300 W for different periods of time (1, 3, 5, 10, 20, 30, 40 and 60 min) were used to prepare samples (“Methods” and Supplementary Information Fig. S1 online). The comparatively higher temperature (115–132 °C) and pressure (287–316 kPa) attained rapidly (within 3 min) in these reactions (Supplementary Information Fig. S2 online) indicate a positive MW heating effect and enhanced reaction rates for the formation of ZnO microcrystals. Heating in MW is known to occur through dipolar polarization and ionic conduction^49^. We attribute the increase in reaction rates to the thermal effect that increase random collisions of thermally excited ions and molecules.

## Results and discussion

**Figure 1:**
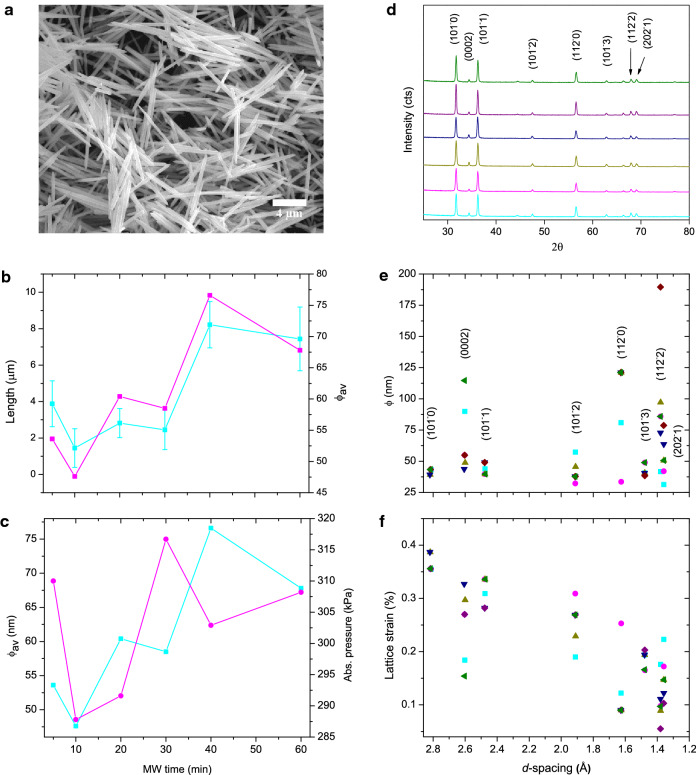
SEM particle shape, size, and XRD crystallite size and strain data plots. (**a**) A representative SEM image of ZnO particles formed. (**b**) Plots of particle length ( ) and ϕ_av_ ( ) of ZnO microparticles and (**c**) ϕ_av_ ( ) and system pressure ( ) against MW time. The particle lengths are measured from SEM images of samples presented in Supplementary Information Fig. S3 online. Error bars show the standard deviation from the mean value of 50 representative particles measured. (**d**) Powder XRD patterns prepared by 5, 10, 20, 30, 40 and 60 min of MW irradiation (from bottom to top). Patterns are shifted vertically for clarity. Scatter plots of (**e**) ϕ and (**f**) lattice strain against *d*-spacings ( —5,  —10,  —20,  —30,  —40, and  —60 min). The crystal planes labelled in (**e**) is applicable to (**f**) as well.

We used three fundamental analysis techniques to examine the nucleation-growth pathways. The final size and shape of the crystals formed were imaged by scanning electron microscopy (SEM) (Fig. 1 and Supplementary Information Fig. S3 online). The size analysis from SEM showed that needle shaped particles with lengths between 1 and 9 μm (for 5–60 min) and widths between 50 and 690 nm were produced. The most significant information on nucleation-growth events was obtained from X-ray diffraction (XRD), Fourier Transform Infrared spectroscopy (FTIR) and SEM-Energy Dispersive X-ray spectroscopy (EDX) data. Specifically, the average crystallite size (ϕ_av_) calculated by averaging out ϕ obtained from all reflections of the diffractogram (ZnO wurtzite phase) showed a direct correlation to the particle size (length and width) (Fig. 1 and Supplementary Information Fig. S4 online) calculated from SEM. The particle length showed a cyclical trend with MW time, initially decreasing then increasing before decreasing again. The ϕ_av_ also followed this trend. The direct correlation suggest that the final particles are formed by the assembly of primary crystallites of 47–77 nm (see Supplementary Table S1 online). On further examining the evolution of size data and pressure with respect to MW time (Fig. 1), it was found that they (ϕ_av_ and particle length) showed an inverse relation to the system pressure above 20 min of MW irradiation, suggesting the crucial role of pressure in the nucleation-growth process.

The evolution of ϕ of the crystal planes during heating (Fig. 1, Supplementary Table S1 online) provides significant clues on the crystallisation process. ϕ does not increase consistently on increasing the MW heating time from 5 to 60 min ruling out nucleation followed by the classical growth phenomenon^50^. Instead, the random ϕ values shown by particles grown with increasing heating time indicates a different growth phenomenon. If the growth had happened through dissolution of Zn^2+^ ions from the side walls (such as {1010} and {1120} planes) of already grown particles followed by added growth along the tips (i.e. {0001} *c*-axes), as reported^51^, the ϕ would not have decreased with increasing MW time. Note also that significant growth modification was observed mainly along {0002} and {1122} planar directions with respect to the pressure changes in the system emphasizing the significant role played by pressure on crystal nucleation and growth.

In order to gain further insight into growth processes, the defect density distribution in crystals was examined by following changes in the lattice micro-strains (Fig. 1). It shows greater strain components associated with the more developed low index planes such as {1010} and {1011}, followed by the {1120} plane, when compared to the other planes. As defects such as dislocations, vacancies, interstitials, substitutions and antisites are manifested by strain-induced diffraction-line broadening^52^, it is arguable that the defects in these samples are more associated with the well-developed planes, that is, associated atomic lattices.

**Figure 2:**
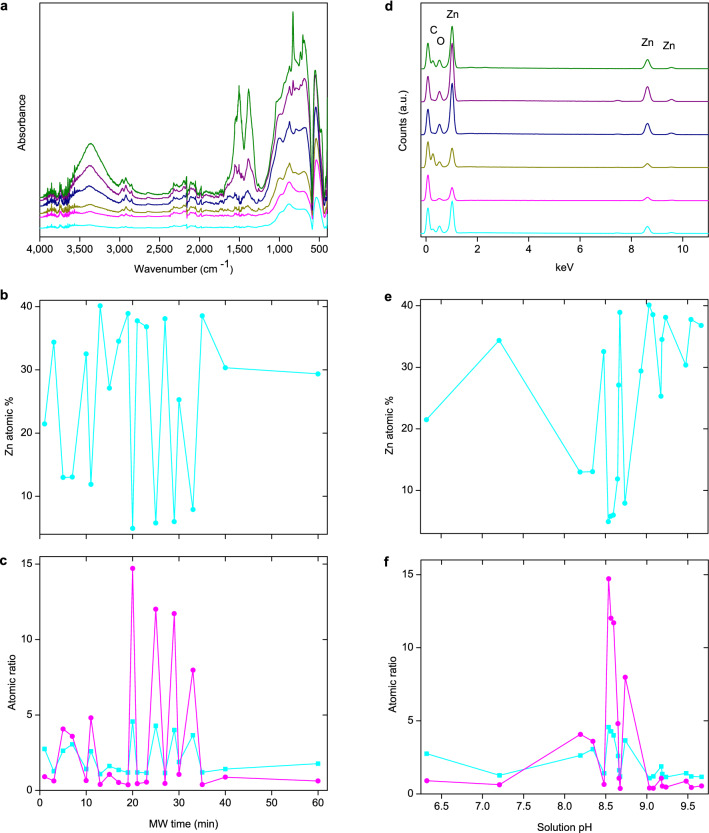
Probing compositional and atomic evolution of samples. (**a**) FTIR spectra of samples prepared by 5, 10, 20, 30, 40 and 60 min of MW irradiation (from top to bottom). The peaks are shifted vertically for clarity. (**b**) Plots of atomic % of Zn and (**c**) O to Zn ( ) and C to Zn ( ) atomic ratios against MW time. (**d**) SEM–EDX patterns of samples discussed. Patterns of 5–60 min samples shifted vertically and stacked from bottom to top. (**e**) Plot of atomic % of Zn and (**f**) O to Zn ( ) and C to Zn ( ) atomic ratios against solution pH. Plotted EDX data are from samples prepared by 1–40 min of MW irradiation with an interval of 2 min, and 60 min. Note that the Zn atomic % is calculated from a 100% total consisting of Zn, O and C.

We then followed nucleation-growth using FTIR of samples following processing. FTIR showed the time dependent evolution of ZnO and associated carbonates, hydroxyls and hydrate species (Fig. 2, see Supplementary Information 5 online for detailed discussions on FTIR and Fig. S5)^53,54^. The decreasing intensity of the carbonate and hydroxyl peaks during heating suggests a time-dependent evolution of the particles. Clearly, their concentration decreases with heating time. Especially, for the higher time samples (30–60 min), three sets of split peaks assignable to the ν_3_(A_u_ + B_u_) modes of carbonates [such as at 1506 and 1340 cm^−1^ (Δν = 166), 1540 and 1374 cm^−1^ (Δν = 146), and 1559 and 1394 cm^−1^ (Δν = 185)], corresponding to three different types of environments were observed. In addition, three broad bands centered around 667–692 cm^−1^, 872–886 cm^−1^ and 941–986 cm^−1^, attributable to the ν_4_(B_u_), ν_2_ out-of-plane bending, and ν_4_(A_u_) modes of carbonates, respectively, were observed^54^. The moderately high Δν_3_ values and the broad ν_2_ and ν_4_ peaks suggest that the carbonate species in the particles mostly take a tridentate/bridging form, involving all three oxygen atoms and are, therefore, probably located between ZnO primary crystallites at the interfacial regions^53–56^. This finding is important since this suggests the significant role played by carbonates in defining the interface of crystallites and their assembly process, which will be discussed in detail later in the paper.

Further examination of the nucleation-growth process was made by considering the changes of Zn atomic %, and atomic ratios of O to Zn and C to Zn with respect to MW heating (Fig. 2). Data for these were derived from SEM–EDX analysis (Fig. 2). Notably, the Zn concentration in particles on increasing the MW time shows a cyclical trend. Further, within this cyclical trend, the Zn content shows a steady increase above pH 9 (whilst maintaining the cyclical trend; Fig. 2) indicating that the particles grow with less organic species in their structure above this pH. This agrees well with the FTIR data. Also, the cyclical trend in Zn content suggests that the nucleation-growth is not a continuous process, again clearly ruling out classical nucleation-growth.

It should, however, be noted that the atomic % of Zn is calculated with respect to that of C and O (Fig. 2). The large cyclical variation in Zn content between samples, thus, emphasizes the dynamism of reaction, resulting in particles with varying organic functionalities (C and O) and Zn, not Zn alone. This also emphasizes the role of carbonates in controlling the particle growth mechanism. This, combining with the crystallite size evolution data (Supplementary Table S1) also emphasize the role of carbonates in restricting the growth of certain planes (e.g. {1120} and {1012}) compared to the growth along other planes ({0002} and {1122}), which is controlled by NH_3_ species.

**Figure 3:**
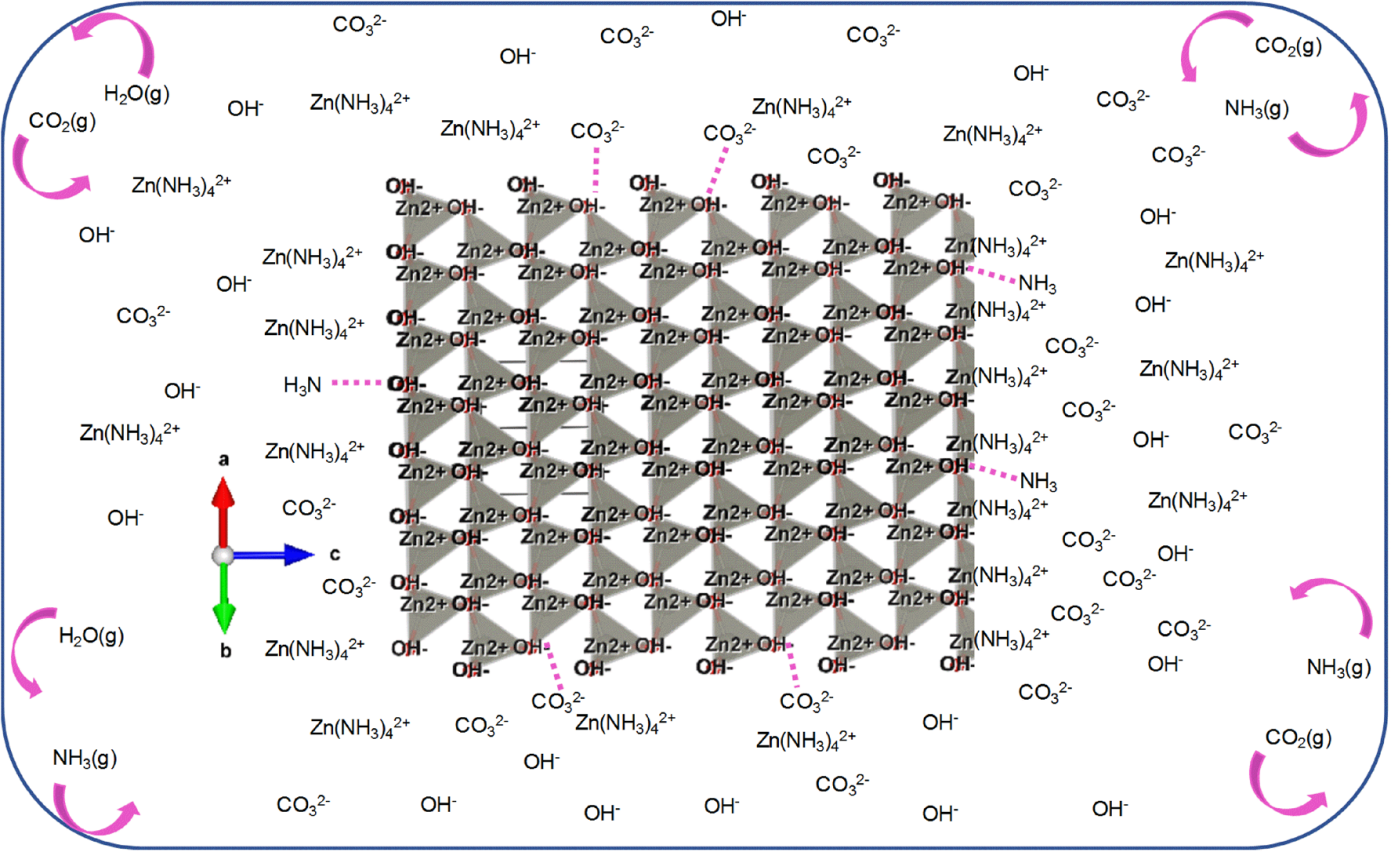
Schematic of a representative PNC, and dynamism of the closed system. 3D *hcp*-like aggregations of Zn^2+^ and OH^−^ ions (without bonding) constitute the PNCs here. Though not drawn for clarity purpose, the Zn^2+^ ions in these PNCs are stabilized by ammonia complexation—as Zn(NH_3_)_4_^2+^. The extent of PNCs is defined by the temporal solution properties, which keep fluctuating throughout the process. PNCs initially co-exist with non-dense regions. Representative ions/species in the non-dense region is shown around the PNC. The dotted lines represent possible hydrogen bonds between species (This is only qualitative representation, not quantitative). Nucleation starts within the PNCs on expulsion of gaseous molecules, especially ammonia complexed with Zn^2+^ ions that stabilizes these PNCs, and carbonates. The growth follows by the aggregate addition of crystallites formed from these PNCs. Dynamism of gaseous molecules is schematically represented.

The proposed nucleation-growth mechanism is discussed hereafter. The decomposition pathway of urea-dissolved-in-water under heat has been documented and is presented in Supplementary Information Fig. S6 online^43^. Under continuous MW heating, initially hydroxides (OH^−^) and bicarbonates (HCO_3_^−^) are formed. The heating also induces de-solvation of ions in solution and ensures they are thermally excited, resulting in a random collision of ions. On continual heating, NH_3_(g), CO_3_^2−^ and CO_2_(g) are formed. As a result, the solubility of Zn^2+^ ions in solution increases, mainly by forming Zn(NH_3_)_4_^2+^ complexes, and the system goes through a supersaturated state with respect to zinc solubility. Over time, the increase in growth-related ionic species in solution, along with the extended time available to organize themselves, drives the Zn^2+^ and OH^−^ ions to attain spatially compact/dense structures—PNCs (Fig. 3, rendered using VESTA software)^57^. Such organization can create a medium range crystalline order among these tightly organized clusters^58^. These clusters remain solvated throughout the heating and allow diffusion of species through its structure—no strict spatial positioning yet. These spatially dense network regions are surrounded by loosely packed regions (interfaces), and, further, randomly distributed regions throughout the system (separation driven by association and interfacial energy dynamics). In the case of ZnO, these dense networks may be organized in *hcp*-like geometries^59^, the boundaries of which are defined by factors such as the ionic strength, pH, the form and concentration/number of all species present and the temperature and the pressure of the system at that point of time. Temporally dynamic addition and exclusion of ions to/from their boundaries will be ongoing throughout the heating in this non-equilibrium system, which is validated by the consistent inverse relation shown by ϕ_av_ and particle size with system pressure above pH 9, or above 20 min of MW irradiation (Fig. 1). This suggests the influence of dissolved ammonia and carbonate species in controlling PNCs’ interface/size and the aggregation process.

Briefly, in the dynamic process, the system vapour pressure increases when parts of the dissolved gases are released. For example, at 30 min, more gaseous molecules are released. Specifically, the NH_3_ molecules complexed with Zn^2+^ ions and carbonates linked at the lateral planar atomic positions situated at the interfaces of the PNCs are released. This will reduce the effective size of the PNCs (crystallites in turn, see XRD crystallize size data in Supplementary Table S1 online). System attains a low vapour pressure when the dynamism of the system forces the gaseous molecules to re-dissolve in the solution. The dissolution of ammonia appears to make notable influence on the crystallite size. Specifically, the length of two crystal planes such as {0002} and {1122} appears to be increased by the dissolution of ammonia. This shows that ammonia dissolution favours zinc ion complexation that places the Zn(NH_3_)_4_^2+^ complexes at these atomic positions increasing atomic density at these planes. This dynamic process continues throughout heating, as evidenced by the pressure-dependent evolution of ϕ. Precisely, the system remains in a temporal and dynamic non-equilibrium state throughout heating, in which, the boundaries of individual PNCs keep rearranging depending on the pressure dynamics and mass transfer to PNC interfaces.

It can be noticed from crystallite size evolution data (Supplementary Table S1) that the planes other than {0002} and {1122} show lesser modification, and especially planes like {1120} and {1012} show little modification above 20 min of reaction. This suggests to the extent of rearrangements happening at the interface of PNCs along different crystal planes. This observation along with the FTIR data indicates to a possible lateral linkage of PNCs, on the complete dissociation of urea, forming laterally extended PNC networks. Crystallite size evolution, EDX and FTIR data, together with pH evolution, suggest that such laterally extended PNC networks are formed from 20 min of MW irradiation. This is consistent with the trends observed substantiating the decisive role played by the smallest components of urea dissociation products (NH_3_, CO_3_^2−^ and CO_2_) in defining the crystallisation process. Below 20 min, prior to the complete dissociation of urea, the particle formation is inconsistent. Above 20 min, when urea is dissociated completely into the smallest components, the PNC-mediated crystallisation and the related trends discussed above becomes consistent. These laterally extended PNC networks thus limits the lateral growth of PNCs. However, the diffusivity of growth units through the PNC networks would still allow certain level of lateral rearrangements. The pressure dynamism, at the same time, causes the PNCs to grow/dissolve at their axial directions more freely.

In order to initiate nucleation, in classical nucleation, the bulk energy term of PNCs should surpass their surface term^3,4,20,60^. In case of ZnO, factors that could favour the bulk term are the mutual affinity of Zn^2+^ and OH^−^ ions, owing to the potential of stabilization by sharing these ions’ high energy orbitals in the subsequent ZnO wurtzite crystal state. As the heating is achieved by MW irradiation, there can be another factor, which is the entropy gain achievable by these organized ions in the PNCs, resonating with the MW field (vibrational entropy, as compared to the configurational entropy)^14^. That is, within the organized cluster form, they could contribute to a favourable entropy term, through their resonant movements with oscillating MW field.

However, these bulk terms do not appear to be sufficient-enough to initiate nucleation during the heating stage. If they had nucleated during heating, like in classical nucleation, the evolution of ϕ should have shown only a progressive trend. In the absence of other factors, the only factor that initiate nucleation is the expulsion of dissolved gaseous molecules. However, when the heating is on, the closed system forces the gas molecules to re-dissolve, making nucleation unfavourable. This dynamic expulsion-dissolution process goes on throughout heating. On stopping irradiation, the temperature and pressure of the system decreases and the dissolved gaseous species (NH_3_ and CO_2_) along with water vapour are expelled irreversibly. This initiates nucleation and the most probable locations for nucleation are the PNCs. Here, the stability of PNCs until the termination of heating is attributed to the Zn(NH_3_)_4_^2+^ complexes stabilizing the Zn^2+^ ions in PNCs. On termination of heating, these zinc-ammine complexes are destabilized, and ammonia is released. This irreversible release forces the PNC structures to nucleate, where the nucleation will start from within the PNCs. The nucleation events stop when these PNCs are fully crystallized by adding growth species from the loosely packed interface region. Here, the boundaries of the formed crystallites are determined by the concentration of growth species and other compatible species, such as carbonates and amines, around the PNCs at the time of stopping heating. The chemisorption of carbonates perturbs the initial crystallisation, creating surface defects. FTIR and the crystallite size evolution data substantiates such perturbation events. Therefore, it is deduced that the defects are mostly associated with the crystallite boundaries. However, the inherent defects can also be created throughout the crystalline structure depending on the temporal system composition at the time of rapid nucleation, that is, at the time of stopping heating, and followed growth events.

In the subsequent growth process, the PNC networks aggregate together (see Supplementary Information Fig. S7 online), and form micron sized particles dictated by factors such as (1) the inherent growth habit of crystals in the absence of other growth modifying agents, and (2) favourable interfacial forces. During aggregation, the crystallites and grains carry chemisorbed species such as OH^−^, HCO_3_^−^ or CO_3_^2−^, depending on the presence of them in solution at that point of time (this depends on the solution pH and pKa; Supplementary Information Fig. S6 online). The relation between dissolution and expulsion of gaseous species and the size and shape evolution of particles, along with the FTIR data suggest that growth occurs by the lateral assembly of crystallites, influenced by the carbonate species, and by their axial assembly dictated by the expulsion of ammonia species. This growth mechanism also explains the formation of a high concentration of defects at the well-developed planes, as evidenced by the XRD micro-strain data. Briefly, the crystallites, as they grow by edge-on (lateral) and head-on (axial) addition between them, the more defects are prone to accumulate at the lateral planes. The higher energy of the lateral planes, due to the presence of more energetically growth-favourable, that is, defective edges, may be the driving force for the lateral assembly process. The dissolved ammonia species in the solution stabilize the inherently energetic axial planes, through zinc-ammine complexes and/or oxygen-ammine hydrogen bonds. The expulsion of *c*-axis stabilized ammonia species initiates the axial aggregation process. At higher pH conditions, CO_2_ formation will be more pronounced and its expulsion results in particles with less organic content. These findings imply that carbonates are important in controlling the crystallite size and on the crystallite aggregation process. Specifically, the carbonates and CO_2_ control the lateral PNC/crystallite growth, assembly and organic content of particles. The absence of evidence of the NH_3_ species in the structure implies that they play key roles in controlling the Zn^2+^ solubility in solution and the *c*-axial growth.

A consistent inverse relation of ϕ_av_ and particle size with the system pressure is observed for samples prepared above 20 min of MW irradiation. This implies that a complete dissociation of urea into its smallest components is required for the system to yield consistent results. Before 20 min, the system pressure is mainly developed from water vapour and partial dissociation of urea. Corroborating results from FTIR, EDX and atomic ratios suggest a lesser availability and activity of carbonates before 20 min. This supports the inconsistent relation between ϕ_av_ and system pressure until 20 min of MW irradiation (See detailed discussions in Supplementary Information 4 and Supplementary Information 8 online).

Further, although the Zn cyclical trend was consistent throughout MW irradiation, a large variation in Zn content was observed in the 20–30 min reaction window (Fig. 2). The examination of the evolution of pH (Supplementary Fig. S4 and Supplementary Information 4 online) and corresponding pKa values (Supplementary Information Fig. S6 online) suggests that the Zn evolution in particles has a direct link to the pH evolution over pressure dynamics. The sequential release of OH^−^, HCO_3_^−^, NH_3_ and CO_2_ with respect to the evolution of pH justifies the large variation between 20 and 30 min of MW irradiation and its direct correlation to a consistently high Zn content above 30 min of reaction time.

## Conclusions

Until this time, every crystallisation study reported has discussed about a classical nucleation/crystallisation or nucleation-growth involving metastable or transient intermediates including prenucleation clusters. No study has reported a cyclical evolution of crystallite size or its constituents, here Zn content, in forming/growing particles that emphasize the possibility of a different crystallisation phenomena in closed reaction systems. We here, by combining coherent data from XRD, SEM–EDX and FTIR showed that the crystallisation of ZnO under MW reaction conditions occurs through a PNC pathway, where the nucleation and followed aggregate addition of PNCs/crystallites lead to final particles. With corroborative evidences, we have also showed that the nucleation commences only when the MW reaction is stopped as opposed to previous reports which all were based on classical nucleation growth or dissolution-growth phenomena for particle formation. We believe that this is a significant new knowledge which could trigger new thinking in this direction.

The importance of this established nucleation-growth mechanism is that this allows the control of materials structure and function by carefully selecting solute concentration, growth controlling chemical additives, temperature, pressure, time of reaction (stopping) and cooling. Thus, this presents a unique method to use closed reaction systems effectively to develop tailored materials of designed structure and functions. This generic predictive method can therefore help improve materials synthesis strategies, and also provide direction for future theoretical and modelling studies. Finally, this also happens to be the first report demonstrating a PNC-mediated nucleation-growth of ZnO under MW irradiation conditions, as opposed to all previous reports that suggested either classical nucleation-growth^3,50^ or dissolution-recrystallisation growth^38,39^.

## Methods

### Reaction set up used for ZnO synthesis

The synthesis was carried out using a modified MW set up. A CEM Technology Discover microwave synthesizer was modified by connecting a reflux condenser to the reaction vessel through a waveguide port to prevent the MWs from escaping (see Supplementary Information Fig. S1 online). The pressure of the system was monitored by connecting the reflux condenser to the pressure gauge, sealing the system, and the temperature was monitored by inserting a probe through the reflux condenser thermometer port. Pressure and temperature data were collected by a Pasco PASPORT Absolute Pressure/Temperature Sensor (model: PS-2146) and recorded using Pasco Capstone software. The addition of the reflux condenser allowed for recovery of lost solvent and prevented excess pressure build-up, extending the possible reaction time, yet conforming to a closed reaction system. The comparatively higher temperature (115–132 °C) and pressure (287–316 kPa) attained rapidly (within 3 min) in these reactions (Fig. S2) indicate a positive MW heating effect and enhanced reaction rates for the formation of ZnO microcrystals. Heating in MW is known to occur through dipolar polarization and ionic conduction^49^. We attribute the increase in reaction rates to the thermal effect that increase random collisions of thermally excited ions and molecules.

### Synthesis of ZnO particles

In a typical experiment, 80 mL aqueous solution containing 5 mM zinc nitrate (Zn(NO_3_)_2_·6H_2_O, 99.0%, Sigma Aldrich, 0.119 g) and 50 mM urea (CO(NH_2_)_2_, 100.0%, Sigma Aldrich, 0.240 g) were placed in a Pyrex round bottom flask and stirred for 10 min and then irradiated at 300 W for 20 min in the MW reactor. Following the MW irradiation, the sample was allowed to cool to room temperature and solution pH was recorded using a Metrohm 632 pH-meter (Switzerland). The precipitate was then separated by centrifugation and washed three times with double distilled water. The sediment was then dried at 60 °C in an air oven before further analyses. Similarly, samples were prepared with different MW times such as 1, 3, 5 up to 40 min, with intervals of 2 min and 60 min, keeping all other parameters unchanged. All syntheses were done using zinc nitrate and urea solutions taken from the same bulk solutions to avoid variation in concentration of solutes between samples.

### Profiling of compositional evolution

Fourier transform infrared spectroscopy (FTIR) was used to profile the compositional evolution of samples, by determining the evolution of IR active functional groups based on their fingerprint absorption properties. FTIR spectra of the powder samples were recorded using a PerkinElmer Spectrum Two FTIR Spectrometer. Average % transmittance data of 20 accumulations were collected between wavenumbers 400–4000 cm^−1^ with a resolution of 1 cm^−1^ for each powder sample and converted to absorbance data.

### Analysis of particle size and shape

FEI Quanta FEG 650 SEM microscope was used for imaging formed particles. The particle lengths and widths were measured from SEM images using ImageJ software^61^. For each sample, the lengths and widths of 50 representative particles were measured and the standard deviation from the mean value of 50 measurements were also presented.

### Probing of atomic evolution in samples

The evolution of atoms such as Zn, C and O with respect to MW heating was examined using SEM–EDX spectroscopic elemental analysis technique. Although EDX is not a strictly quantitative tool for the elemental analysis for determining O and C concentration due to the possible environmental contamination, the comparative data presents a clear trend which agrees well with the FTIR results. This substantiates the reliability of elemental analysis in this study.

### Determination and profiling of crystallite size and lattice strain evolution

Room temperature powder XRD patterns of the samples were recorded using a Panalytical X-ray diffractometer in the diffraction angle range of 2θ 20°–80° using CuKα radiation (wavelength 1.541874 Å) produced at a voltage of 45 mV and current 40 mA. The XRD data were used to determine the crystallite size and lattice micro-strain by line profile fitting analysis. The XRD profile fitting was carried out using the X'Pert HighScore software to decompose the powder pattern to extract accurate peak parameters^62^. The software deconvolutes the overlapped peaks by fitting with a Pseudo-Voigt profile function, which is the weighted mean between a Lorentzian and a Gaussian function. The full width at half maximum (FWHM; B in radians) of the peak obtained from a Lorentzian fit was then used to calculate the average crystallite size (ϕ) using the Scherrer formula; ϕ = Kλ/(B cos θ). The FWHM from Gaussian fit was used for calculating the mean lattice strain using the tangent formula; Lattice strain (mean lattice distortion) = B/(4 tan θ), where B describes the structural broadening, which is the difference in integral profile width between a standard and the unknown sample (Eqs. 1 and 2):

1 $${\text{B}}\left( {{\text{size}}} \right) = {\text{B}}_{{{\text{obs}}}} {-}{\text{B}}_{{{\text{std}}}}$$

2 $${\text{B}}\left( {{\text{strain}}} \right) = \left( {{\text{B}}_{{{\text{obs}}}}^{{2}} {-}{\text{B}}_{{{\text{std}}}}^{{2}} } \right)^{{{1}/{2}}}$$

An instrumental broadening (B_std_) of 2θ 0.0455 obtained by measuring the broadening of a standard silicon sample (Panalytical) was used for all calculations (see Supplementary Information Fig. S1 online).

## Supplementary information

Supplementary Information.

## Data Availability

All relevant data are presented either in the article or in “Supplementary information” online.
