# Supplementary material for: A conceptual change in crystallisation mechanisms of oxide materials from solutions in closed systems
Source: Sci Rep. 2020 Oct 27;10:18414. doi: 10.1038/s41598-020-75241-z (PMC7592049; doi:10.1038/s41598-020-75241-z)
Supplement: Supplementary file 1 — Supplementary Information. [file 41598_2020_75241_MOESM1_ESM.pdf]

Supplementary Information

A conceptual change in crystallisation mechanisms of oxide materials from solutions in closed systems

Sibu C. Padmanabhan,[1,2,3]* Timothy W. Collins,[3] Suresh C. Pillai,[4,5] Declan E. McCormack,[6] John M. Kelly,[2] Justin D. Holmes, [1,2,3] Michael A. Morris[1,2]*

[1]Advanced Materials and BioEngineering Research (AMBER) Centre, Trinity College Dublin, College Green, Dublin 2, Ireland.

[2]School of Chemistry, Trinity College Dublin, College Green, Dublin 2, Ireland.

[3]School of Chemistry, University College Cork, College Road, Cork, T12 YN60, Ireland.

[4]Centre for Precision Engineering, Materials and Manufacturing Research (PEM), Institute of Technology, Sligo, F91 YW50, Ireland.

[5]Nanotechnology and Bio-Engineering Research Group, Department of Environmental Science, Institute of Technology Sligo, F91 YW50, Ireland.

[6]School of Chemical & Pharmaceutical Sciences, Technical University Dublin, City Campus, Kevin Street, Dublin 8, D02 HW71, Ireland.

Email: *chullans@tcd.ie, morrism2@tcd.ie

SI1. MW set up and XRD instrumental broadening

Figure S1 presents photograph of the modified MW set up employed for this work. The CEM Technology Discover system utilizes a self-tuning microwave cavity, which ensures that the reaction vessel is uniformly exposed to microwaves from every direction. In addition, the penetration depth of microwaves in water at 95 °C is 5.7 cm, which is greater than the diameter of the reaction vessel and, therefore, uniformly providing energy to the reagents.

To estimate the effect of MWs on the process, MW absorptivity of water, urea and their decomposition products must be considered. Water is only a medium MW absorber with tan $\delta = 0.123$, where tan δ is the dielectric loss tangent; a ratio between dielectric loss ε'', representing the efficiency with which electromagnetic radiation is converted into heat and dielectric constant ε', describing the ability to be

polarized by the electric field such that tan δ = ε″ / ε′. This loss factor provides a measure of the ability of a material to convert electromagnetic energy into heat at a given frequency and temperature. The tangent loss is similar for water and urea[1].

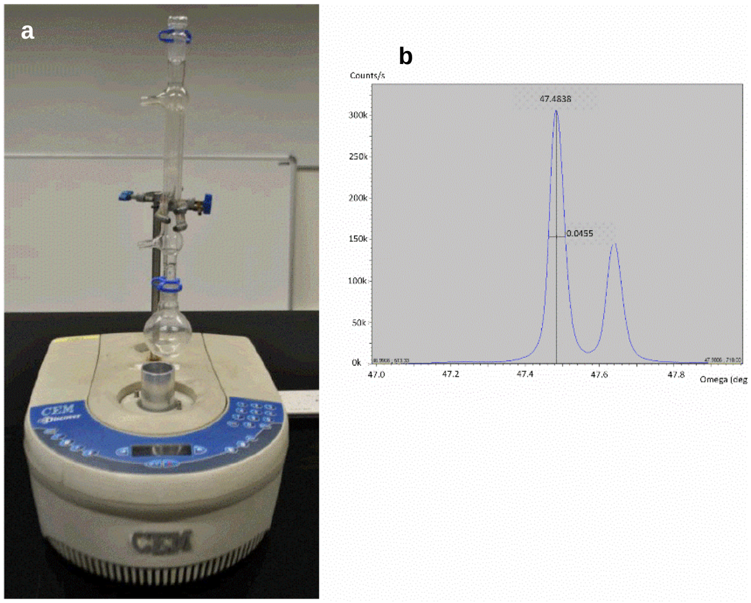

Supplementary Figure S1. Experimental details (a) Picture of the modified MW set up used. (b) XRD pattern showing the instrumental broadening obtained from a standard silicon sample.

SI2. System temperature and pressure profiles

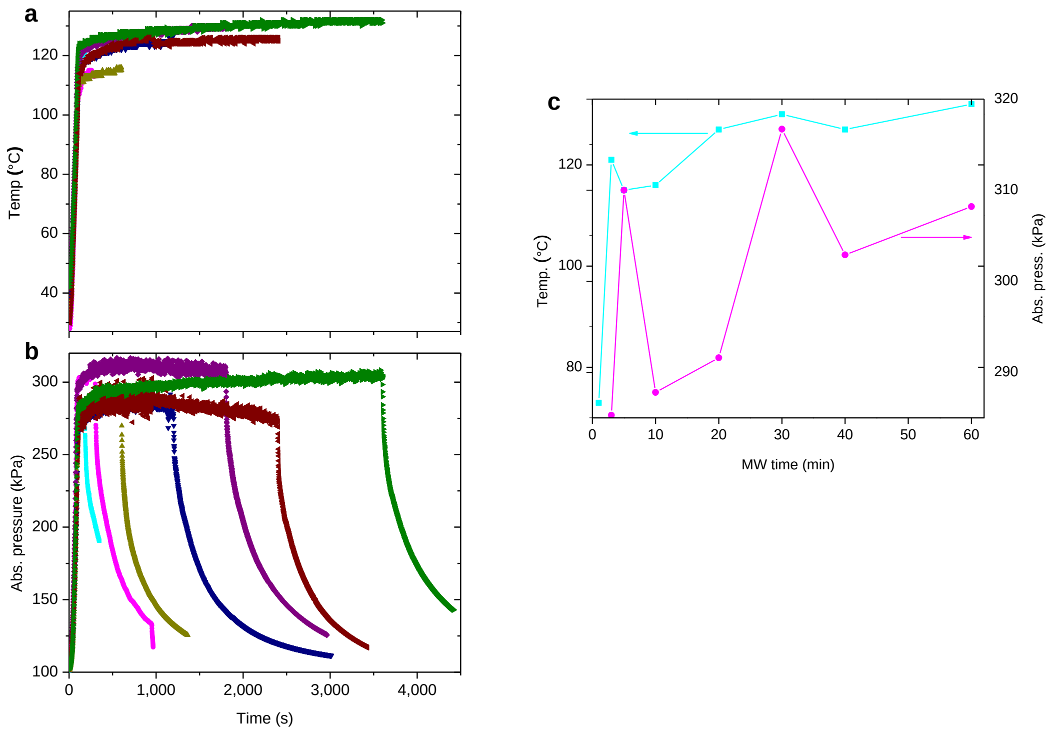

Supplementary Figure S2. The evolution of temperature and pressure of the systems on increasing MW time. Profiles of (a) temperature and (b) absolute pressure against MW time. (■ - 3, ● - 5, ▲ - 10, ▼ - 20, ◆ - 30, ◄ - 40, ► - 60 min). (c) Plots of highest temperature and pressure attained by systems (1, 3, 5, 10, 20, 30, 40 and 60 min) against MW time.

SI3. SEM of samples

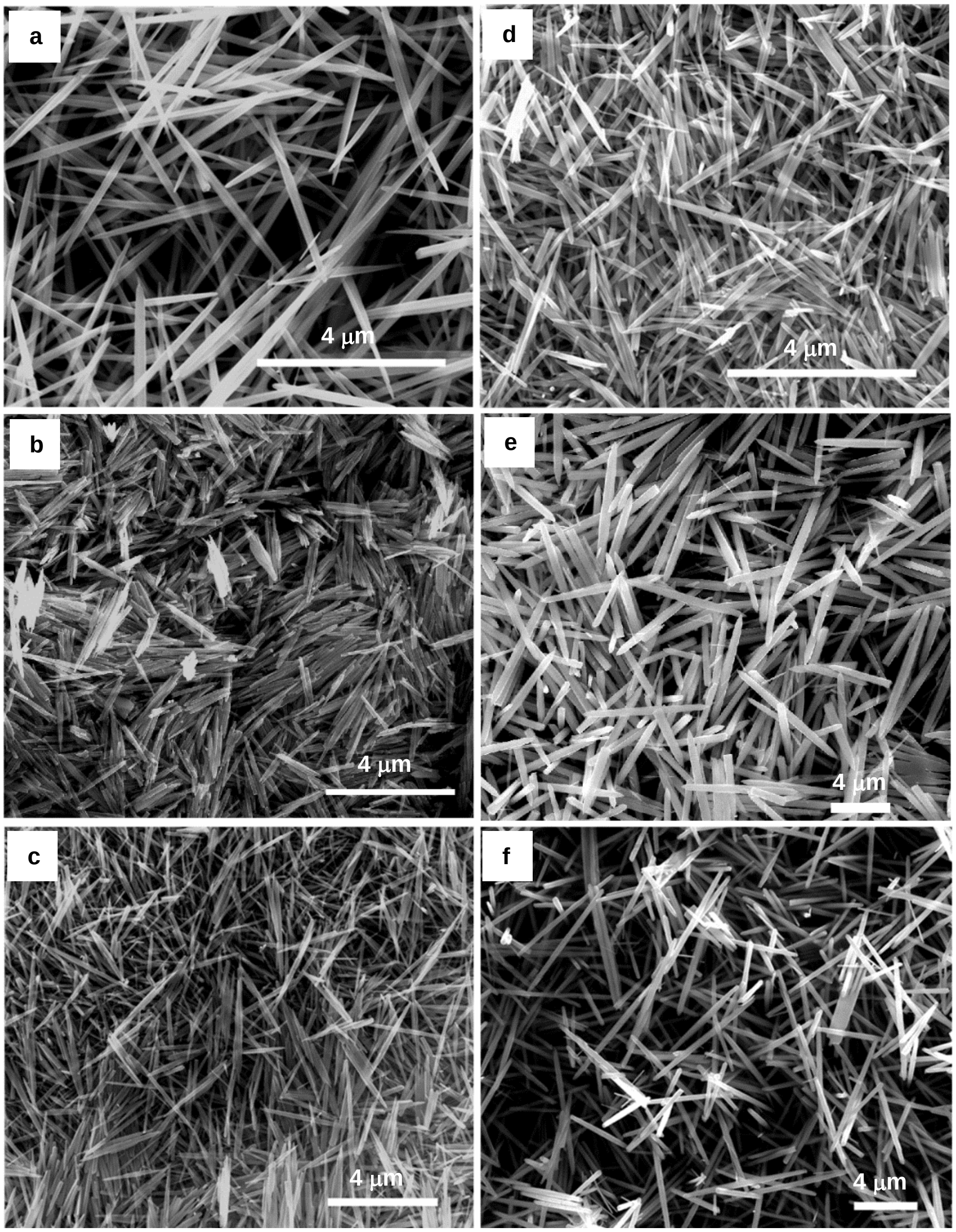

Supplementary Figure S3. Probing particle shape and size evolution using SEM. (a-f) SEM images of particles obtained from MW irradiation for 5, 10, 20, 30, 40 and 60 min respectively.

SI4. Size, elemental analysis data and system pressure with respect to MW irradiation time

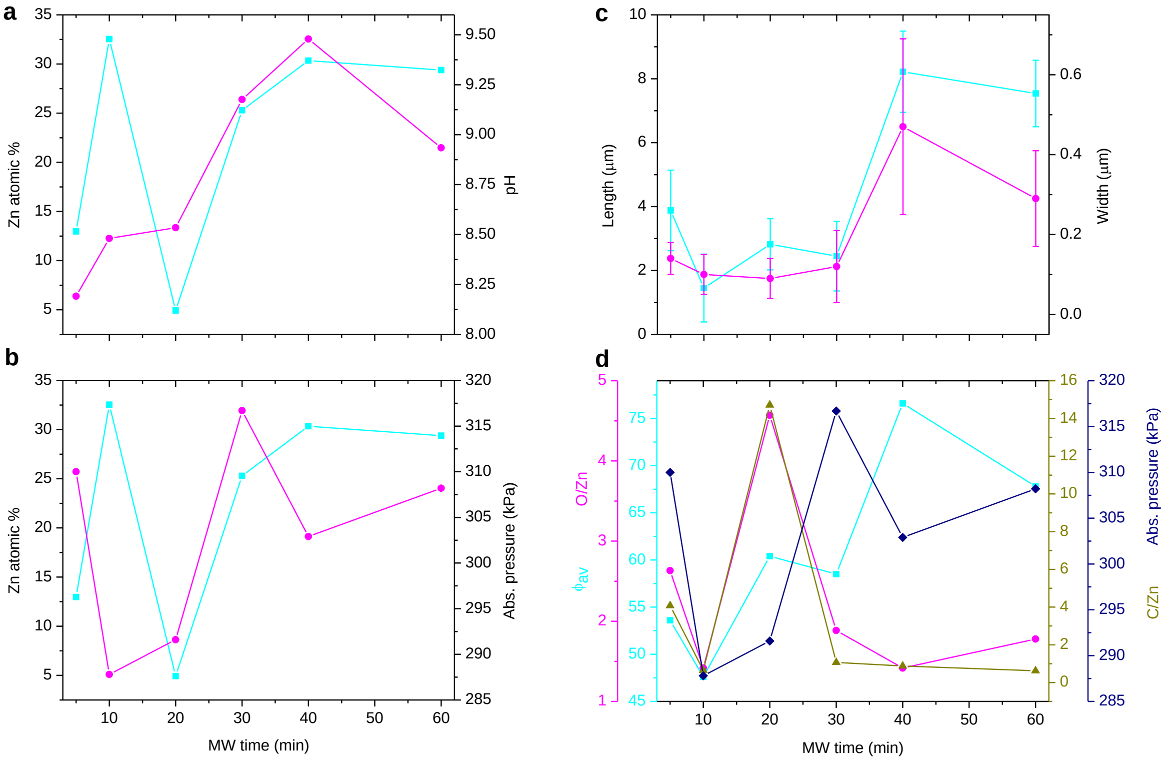

Supplementary Figure S4. (a) Plots of Zn atomic % (—■—) and pH (—●—), (b) Zn atomic % (—■—) and system pressure (—●—), (c) particle length (—■—) and width (—●—), (d) ϕ_{av} (—■—), O/Zn (—●—), C/Zn (—▲—) and system pressure (—◆—), all against MW time.

Before 20 min, the complete dissociation of urea into the smallest components is not facilitated as per the FTIR and EDX data. Therefore, below 20 min of MW irradiation, as the number of growth units are less, the resultant inhomogeneity of the system would lead to competing and inhomogeneous crystallite assembly driven growth process producing particles with a wide size range. The complete dissociation of urea at higher MW times would lead to more of a homogeneous-like PNC system and resultant particles will be more homogeneous with small size distribution. This is the reason for the larger error bar for the

lower MW times samples. Nevertheless, if we consider the particle length values and crystallite size evolution with respect to MW time, we can see a similar and much clearer trend in both. Further, the standard deviation from the mean values decreases considerably on increasing the MW time, making the trend more prominent.

SI5. FTIR data of samples in detail

FTIR spectroscopy is a bulk non-destructive characterization technique to determine the structures of samples, by determining the IR active functional groups based on their fingerprint absorption properties and their concentrations (from peak heights). The ZnO growth is profiled based on their presence, mode of attachment and concentrations determined from FTIR data. The FTIR spectra of all the samples showed varying amounts of characteristic vibrational peaks corresponding to O-Zn-O, CO_3^{2-}, HCO_3^- and OH groups (Fig. 2 in the article). The peak centered between 506 and 565 cm^{-1} is attributable to the O-Zn-O phonon vibrations. The particles formed after 5 min irradiation show two peaks centered at 469 and 576 cm^{-1} with a small peak at 507 cm^{-1}. The 469 cm^{-1} peak diminishes on increase of MW time, which is attributable to the Zn-OH translation mode[2]. Other than this, the peaks at 831, 949 and 1043 cm^{-1} can also be assigned to Zn-OH deformation, liberation and bending modes respectively[3,4]. All these peaks are only visible for the 5 and 10 min samples, and especially, the sharp peaks of Zn-OH modes seem to mask most of the peaks corresponding to carbonates in the 5 min sample. On increase of MW irradiation time, the peak at 576 cm^{-1} shifts to lower wavenumbers (538 cm^{-1} for the 60 min sample) and broadens. The broadening indicates the possible disturbance in the lattice causing uneven O-Zn-O atomic distances[5]. The possible reason could be the influence of the defective structural environment such as the presence of interstitials (Zn_i, O_i) and vacancies (V_{Zn}, V_O) in the ZnO lattice.

The internal modes of CO_3^{2-} species

The internal modes of the free carbonate ions (D_{3h} point group symmetry) are as follows, $\nu_1(A'_1)$ symmetric C-O stretching mode; $\nu_2(A''_2)$ out-of-plane O-C-O bending mode; $\nu_3(E')$ C-O stretching mode,

and $\nu_4(E')$ in-plane O-C-O bending mode. Due to the site symmetry of C of the CO_3^{2-} ions in the ZnO wurtzite lattice, the degeneracy of both the ν_3 and ν_4 modes are lifted and, hence, two $\nu_3(2A)$ and $\nu_4(2A)$ components will appear in the IR spectrum. Additionally, each of the six non-degenerate modes (A species) of the CO_3^{2-} ion could split into two IR active components A_u and B_u under C_{2h} symmetry, as a result of the correlation field splitting. Furthermore, when more than one carbonate ion exists in the lattice, more sets of the internal modes will appear in the spectra, corresponding to the number of crystallographically different carbonate ions.

The splitting of ν_3 and ν_4 modes in the current samples

The lifting of degeneracy of the unperturbed free carbonate ions on coordination to Zn moieties (on changing the symmetry) will be reflected as splits in its ν_3 and ν_4 modes as (A_u + B_u) components around its uncoordinated frequency of 1400 cm^{-1} and 770 cm^{-1} respectively. When more than one carbonate ion exists in the particle (at the crystallite or grain boundaries or even associated within the lattice), more sets of the internal modes can appear in the spectra, corresponding to the number of crystallographically different carbonate ions[4]. The frequency difference upon coordination, $\Delta\nu$, is a measure of the interaction with the surface. Hence, the $\Delta\nu$ values calculated can be used to assign coordinated carbonate to mono-, bi-, tri and bridged configurations ($\Delta\nu_3$ = <100 cm^{-1}, 100 < $\Delta\nu_3$ < 300 cm^{-1} and $\Delta\nu_3$ > 400 cm^{-1}, respectively).

Figure S5 presents FTIR spectra of particles prepared by MW irradiation for 20, 30, 40 and 60 min. The spectral window from 1250 – 4000 cm^{-1} is presented to clearly show the low intense carbonate peaks. The presence of carbonates in the samples are represented by the peaks that appear at 879 ± 7 cm^{-1} ($\nu_2(A''_2)$ out-of-plane deformation/bending of CO_3^{2-}), the split peaks at 1506 and 1382 cm^{-1} (corresponding to the unidentate $\nu_3(A_u + B_u)$ (CO_3^{2-}) asymmetric stretching), and at 1551 and 1350 cm^{-1} (corresponding to the bidentate $\nu_3(A_u + B_u)$ (CO_3^{2-}) asymmetric stretching), the peak at 667 cm^{-1} (corresponding to ν_4 (E') in-plane CO_3^{2-} bending), and those at 737 and 707 cm^{-1} attributable to the (A_u + B_u) components of the ν_4 mode (Fig. 2a in main text & Fig. S5)[4,6]. These peaks are clearly evident only for the 5 and 10 min MW

irradiated samples. Further, the 5 and 10 min samples show a broad, low-intense peak between 1641 and 1708 cm^{-1} suggesting the presence of some bicarbonates in the structure[4,6]. The CO_3^{2-} species in the structure are believed to be originated from the decomposition of urea. The decomposition of urea to HCO_3^- and then to CO_3^{2-}, or the dissolution of the decomposed CO_2 in solution can both be responsible for the intercalation of this species in the ZnO structure. It also indirectly indicates that the system goes through a higher pH at the time of reaction than the measured pH when the solution is cooled down to RT. Apart from these, a sharp absorption peak at 1387 cm^{-1} in the 5 and 10 min samples suggests the presence of Zn bound NO_3^- species which on increasing the irradiation time seems to disappear. Although the $\Delta\nu_3$ values obtained in these samples are not high enough, the $\nu_3(A_u + B_u)$ modes of carbonates in three different types of environments are observable; which are at 1506 and 1340 cm^{-1} ($\Delta\nu$ = 166), 1540 and 1374 cm^{-1} ($\Delta\nu$ = 146), and 1559 and 1394 cm^{-1} ($\Delta\nu$ = 185)). The presence of carbonates in particles even after the extended MW processes suggests that they are stable against desorption, and hence can be assumed to form complexes using stable multi-dentate or bridged co-ordinations.

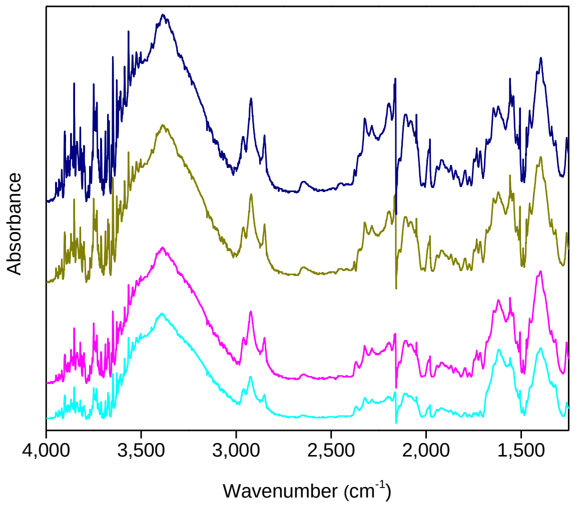

Supplementary Figure S5. FTIR spectra of samples prepared by 20, 30, 40 and 60 min MW irradiation (stacked from bottom to top). Spectral window from 1250 - 4000 cm^{-1} is presented for better visibility of the low-intense carbonate peaks.

Further up in the spectra, the small peaks at 2201, 2286 and 2323 cm^{-1} suggest that the particles are adsorbed with small quantities of CO_2 (ν_3 asymmetric stretch) probably on their surfaces. The small peaks at 2851 and 2922 cm^{-1} corresponds to C-H stretching[5]. Notably, the C-H peaks are only observable for samples prepared at and above 10 min of MW irradiation suggesting that they may have formed as a side product of the chemical reaction. The broad absorption peaks that appear in the 3000 - 3500 cm^{-1} region suggest the presence of OH or NH groups in the structure, more preferably OH in the form of zinc hydroxide and hydrogen bonded hydroxyl and hydrates species in lower MW irradiated samples (5 and 10), and hydrogen bonded hydroxyls in the longer time MW irradiated samples (20 and above)[5]. The presence of extensive hydrogen bonding makes this band broad, which indicates the possible presence of hydrogen bonded H_2O, OH or H species in the structure. These can be at the crystallite and grain boundaries and defect sites or as hydrogen bonded to the bridged CO_3^{2-} species. The small discrete peaks above 3500 cm^{-1} can be attributed to OH groups attached to different chemical environments. The peaks in the range 3500 - 3600 cm^{-1} suggest the presence of bridging OH groups exhibiting additional electrostatic interactions to adjacent oxygens[7]. The peaks between 3600 and 3650 cm^{-1} suggest the presence of free bridging OH groups[7]. The peaks around 3720 cm^{-1} may indicate the presence of OH/NH groups attached to the defect sites and those around 3740 cm^{-1} may correspond to the OH/NH groups at terminal lattice sites[7].

SI6. Chemical reactions occurring under the MW process and the pKa values of reactions

Looking at the chemical reactions, the pKa values of reactions (Fig. S6) and the experimental data; the extent of MW irradiation and pH appear to be the deciding factors of the nucleation-growth process. The composition of the chemical species (urea and its decomposition products) keep changing in the solution, owing to sequential decomposition events, and the pH increases on increasing MW reaction time. When the MWs are stopped and the temperature and pressure drop, the dissolved gaseous molecules (CO_2 and NH_3) start expelling from the system. This will force the system that maintains its supersaturation until

that time to undergo nucleation and follow up growth. During such a growth process, the species available at the interface of crystallites and grains at that phase of the reaction can also become integrated into the particle structure dictated by the interfacial and thermodynamic compatibility. By comparing all experimental data, it can be assumed that by increasing the MW time, hence, on increasing the solution pH, the particle nucleation-growth process undergo some refinement in terms of the Zn, O and C content and the crystal structure is refined in terms of the organic content, as suggested by FTIR and EDX; *i.e.* the organic content appears to be reduced on increasing the MW time. This refinement may be driven by the pH/pKa defined speciation events, where the gaseous species like ammonia and CO_2 may be formed, dissolved in the solution due to the closed nature of the system, and expelled when the MW irradiation is stopped.

$$H_2NCONH_2 + H_2O \longrightarrow H_2NCO_2^- + NH_4^+ \qquad (1)$$

$$H_2NCO_2^- + 2H_2O \longrightarrow NH_4^+ + HCO_3^- + OH^- \qquad (2)$$

$$Zn^{2+} + nNH_{3(aq)} \rightleftharpoons Zn(NH_3)_n^{2+} \qquad (3)$$

$$NH_4^+ \rightleftharpoons NH_3 + H^+ , \qquad pKa = 9.24$$

$$NH_2CO_2H \rightleftharpoons NH_2CO_2^- + H^+ , \qquad pKa = 5.8$$

$$HCO_3^- \rightleftharpoons CO_3^{2-} + H^+ , \qquad pKa = 10.3$$

Supplementary Figure S6. Chemical reactions occurring under the MW process and the pKa values of reactions.

Tapering of particles to form needle shaped ends

The tapering of the particles at the ends of the rods are also dictated by the Zn-ammine complexation and the release of ammonia. Briefly, after the fast nucleation and growth processes, the excess Zn available in the solution as Zn-ammine complexes will sequentially be deposited at the bare (no presence of

stabilizing groups) high energy *c*-axial planes. To reduce its energy, the *c*-axes of particles will be grown further by atomic deposition of Zn and OH, and as the concentrations of these species are limited, the system tries to achieve the highest stability structures possible by forming tapered ends.

SI7. Schematic of crystallites formed from PNCs

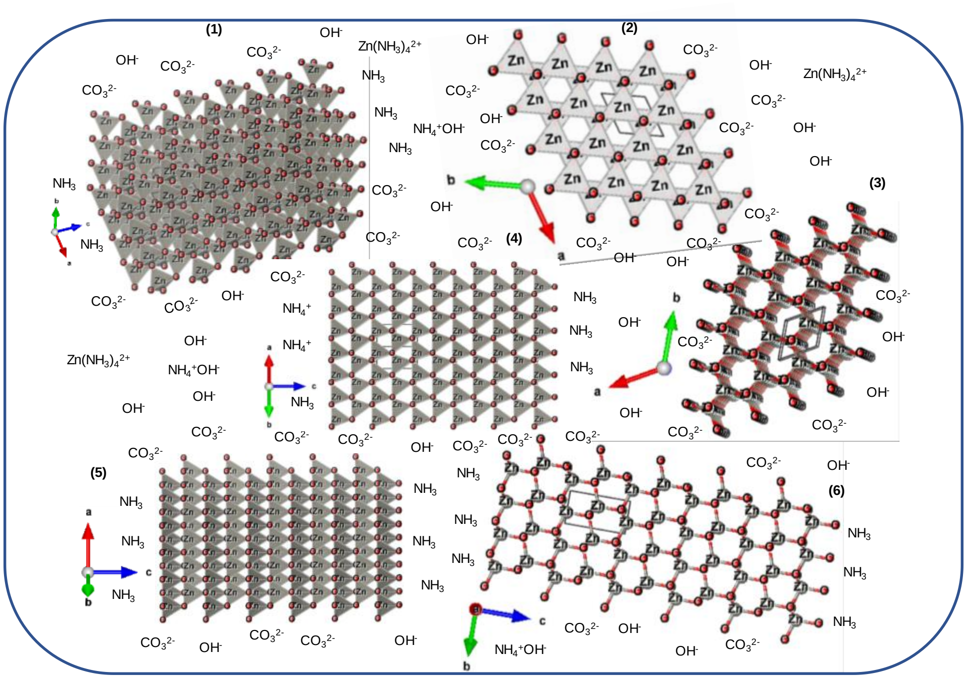

Supplementary Figure S7. Schematic representation of the crystallites formed before aggregation of them to form the final ZnO particles. In the six representative crystallites drawn, 1, 2, 4 and 5 are drawn in polyhedral form and 3 and 6 in ball-and-stick form, drawn at different viewing angles for a better understanding. The CO_3^{2-} ions in the solution influence the growth of particles by influencing mainly the lateral assembly process. The ammonia gas produced will be re-dissolved in solution as $Zn(NH_3)_4^{2+}$ complexes or as $NH_4^+OH^-$, or stabilized by hydrogen bonding to the crystallite boundaries. Experimental evidence and growth patterns suggest that the Zn and O terminated *c*-axial planes are the preferred

stabilization points of ammonia, thereby also influencing the axial growth process on their expulsion, *i.e.* on stopping the MW irradiation. Rendered using VESTA software.

SI8. Crystallite size data of all the crystal planes from XRD

Crystal plane	d (Å)	ϕ (nm)	d (Å)	ϕ (nm)	d (Å)	ϕ (nm)
	5 min		10 min		20 min	
{1010}	2.812	43.4	2.814	43.4	2.818	39.2
{0002}	2.602	89.9	2.603	54.9	2.6	48.8
{1011}	2.476	43.9	2.478	39.7	2.476	39.7
{1012}	1.911	57.3	1.912	32.2	1.91	45.6
{1120}	1.626	80.9	1.625	33.5	1.625	121.1
{1013}	1.477	40.6	1.477	48.9	1.478	40.6
{1122}	1.379	41.7	1.379	85.9	1.379	97.2
{2021}	1.359	31.3	1.359	42	1.359	50.6
ϕ_{av} (nm)		53.6		47.6		60.4

Crystal plane	d (Å)	ϕ (nm)	d (Å)	ϕ (nm)	d (Å)	ϕ (nm)
	30 min		40 min		60 min	
{1010}	2.82	39.2	2.817	43.4	2.816	43.4
{0002}	2.606	43.7	2.603	54.9	2.603	114.7
{1011}	2.478	49	2.478	49	2.474	39.7
{1012}	1.913	37.8	1.912	37.8	1.912	37.8
{1120}	1.627	121.1	1.627	121.1	1.627	121.1
{1013}	1.479	40.5	1.478	38.4	1.478	48.9
{1122}	1.38	72.8	1.38	189.6	1.379	85.9
{2021}	1.359	63.6	1.36	78.7	1.358	50.6
ϕ_{av} (nm)		58.5		76.6		67.8

Supplementary Table S1. Crystallize size data derived from XRD.

As ϕ calculated are averaged values from millions of crystallites, despite not so large changes between crystallite sizes (ϕ_{av}), the change from 53.6 to 47.6, then to 60.4, then to 58.5, then to 76.6 and to 67.8 nm should be considered as significant indicative changes between samples. Further, the evolution of ϕ of different crystalline planes with respect to MW time provide critical information about the crystallization process. Notably the planes such as {1120} and {1012} show little growth above 20 min of MW irradiation. At the same time, the {0002} and {1122} planes show significant change with respect to MW irradiation. The notable change in crystallite sizes of these planes when the other planes remain unchanged or slightly changed provide a strong indication to the potential interfacial reactions happening around the PNCs. This would only be possible if the nucleation-growth would happen only through a PNC pathway. If there was initial nucleation at some point of the reaction through classical nucleation, then the only possibility would be a normal crystallite growth in every direction or aggregate growth. In that situation the identical crystallite size figures of {1120} and {1012} planes would be questionable, as one would expect a normal growth of crystallites.

Crystallite size evolution, together with EDX, FTIR and pH data suggest that the crystallization process becomes consistent above 20 min of MW irradiation. All data indicate to an incomplete dissociation of urea below 20 min. Above 20 min, as urea is dissociated completely into its smallest components, the system becomes homogeneous-like and the PNC-mediated crystallization becomes more consistent. Specifically, above 20 min, the limited growth of lateral crystal planes indicates to a possible lateral linkage between PNCs. However, as PNCs allow diffusion of growth units through them, although limited, certain lateral planes still show limited modifications.

Thus, the crystallization process, despite through PNC mediated mechanism, show a clear division above and below 20 min of MW irradiation. Below 20 min, the PNCs form, then nucleation and follow up aggregation-growth occurs on stopping the MW reaction. Above 20 min, according to all available data, the complete dissociation of urea, the homogeneous-like nature of the system and the time available would lead to a possible lateral linkage of PNCs. On stopping MW reaction, these laterally linked PNCs

nucleate and get locked in and a simultaneous growth occurs through head-on aggregation forming final particles.

References

1 Yadav, V., Kumar, A., Sharan, S. & Sinha, A. K. Analyses of dielectric properties of fertilizers (urea and diammonium phosphate) in aqueous solution at different temperatures in microwave frequency. *Int. J. Phys. Sci.* **5**, 2466-2470 (2011).

2 Kloprogge, J. T., Hickey, L. & Frost, R. L. FT-Raman and FT-IR spectroscopic study of synthetic Mg/Zn/Al-hydrotalcites. *J. Raman Spectrosc.* **35**, 967-974, doi:10.1002/jrs.1244 (2004).

3 Piva, D. H. *et al.* Antibacterial and photocatalytic activity of ZnO nanoparticles from $Zn(OH)_2$ dehydrated by azeotropic distillation, freeze drying, and ethanol washing. *Advanced Powder Technology* **28**, 463-472, doi:https://doi.org/10.1016/j.apt.2016.11.001 (2017).

4 Stoilova, D., Koleva, V. & Vassileva, V. Infrared study of some synthetic phases of malachite ($Cu_2(OH)_2CO_3$)–hydrozincite ($Zn_5(OH)_6(CO_3)_2$) series. *Spectrochim. Acta A: Mol. Spectrosc.* **58**, 2051-2059, doi:https://doi.org/10.1016/S1386-1425(01)00677-1 (2002).

5 Smith, B. C. *Fundamentals of Fourier Transform Infrared Spectroscopy*. 2nd edn, (CRC Press, Boca Raton, Florida, 2011).

6 Scheetz, B. E. & White, W. B. Vibrational spectra of the alkaline earth double carbonates. *Am. Mineral.* **62**, 36-50 (1977).

7 Auerbach, S. M., Carrado, K. A. & Dutta, P. K. *Handbook of Zeolite Science and Technology*. (Marcel Dekker, New York, 2003).
